# Excluding pulmonary embolism in primary care using the Wells-rule in combination with a point-of care D-dimer test: a scenario analysis

**DOI:** 10.1186/1471-2296-11-64

**Published:** 2010-09-13

**Authors:** Wim AM Lucassen, Renée A Douma, Diane B Toll, Harry R Büller, Henk CPM van Weert

**Affiliations:** 1 Academic Medical Centre, Department of General Practice, Amsterdam, The Netherlands; 2 Academic Medical Centre, Department of Vascular Medicine, Amsterdam, The Netherlands

## Abstract

**Background:**

In secondary care the Wells clinical decision rule (CDR) combined with a quantitative D-dimer test can exclude pulmonary embolism (PE) safely. The introduction of point-of-care (POC) D-dimer tests facilitates a similar diagnostic strategy in primary care.

We estimated failure-rate and efficiency of a diagnostic strategy using the Wells-CDR combined with a POC-D-dimer test for excluding PE in primary care.

We considered ruling out PE safe if the failure rate was <2% with a maximum upper confidence limit of 2.7%.

**Methods:**

We performed a scenario-analysis on data of 2701 outpatients suspected of PE. We used test characteristics of two qualitative POC-D-dimer tests, as derived from a meta-analysis and combined these with the Wells-CDR-score.

**Results:**

In scenario 1 (SimpliRed-D-dimer sensitivity 85%, specificity 74%) PE was excluded safely in 23.8% of patients but only by lowering the cut-off value of the Wells rule to <2. (failure rate: 1.4%, 95% CI 0.6-2.6%)

In scenario 2 (Simplify-D-dimer sensitivity 87%, specificity 62%) PE was excluded safely in 12.4% of patients provided that the Wells-cut-off value was set at 0. (failure rate: 0.9%, 95% CI 0.2-2.6%)

**Conclusion:**

Theoretically a diagnostic strategy using the Wells-CDR combined with a qualitative POC-D-dimer test can be used safely to exclude PE in primary care albeit with only moderate efficiency.

## Background

Pulmonary embolism (PE) has an estimated annual incidence of 23 cases per 100.000 persons [1]. Because PE is potentially life-threatening, immediate diagnosis and management is essential. As primary care physicians lack accurate diagnostic tools, all patients have to be referred, often with all due speed to secondary care in case PE is suspected. However in 75-95% of these referred patients PE subsequently is excluded [2-4]. Several management-studies in secondary care have demonstrated that PE can be excluded safely in patients with a low (<2) or unlikely (≤4) clinical probability according to the clinical decision rule (CDR) as developed by Wells et al.(Table 1), combined with a normal D-dimer test result (both quantitative and qualitative D-dimer tests) [5-8]. The introduction of easy-to-use rapid point-of-care (POC) D-dimer tests makes it possible to exclude PE safely in the primary care setting, using a diagnostic work-up similar to that in secondary care thereby avoiding unnecessary referrals.

**Table 1:** Wells clinical decision rule.

Variable	Points
Clinical signs and symptoms of DVT (minimum of leg swelling and pain with palpation of the deep veins)	3.0

Alternative diagnosis less likely than PE	3.0

Heart rate > 100/min	1.5

Immobilization (>3 days) or surgery in the previous 4 weeks	1.5

Previous PE or DVT	1.5

Haemoptysis	1.0

Malignancy (receiving treatment, treated in the last 6 months or palliative)	1.0

Clinical probability of PE:

Unlikely ≤4 points

Likely >4 points

Low <2 points

Intermediate 2-6 points

High >6 points

Abbreviations: DVT, deep vein thrombosis; PE, pulmonary embolism

Qualitative POC D-dimer tests do not need additional equipment or calibration, are ready to use, cheap, utilize capillary or venous blood and can be done in-and outside the clinic. They can be interpreted within 10 minutes as either positive or negative which make the tests suitable for use in primary care. Questions have been raised however about the sensitivity of the tests ranging from 80-100% in different studies [7,9-13].

To our knowledge a management-study with a diagnostic strategy using a CDR in combination with POC-D-dimer test for excluding PE has not been performed in primary care although this approach was successfully used in the setting of suspected deep vein thrombosis (DVT) [14]. We performed a scenario-analysis to calculate the expected results of such a management strategy in patients referred by their primary care physician for suspected PE. Because exclusion of PE is based on the probability score of the Wells rule combined with the result of a qualitative D-dimer test we aimed to calculate a safety-threshold by varying the cut-off value of the Wells-rule.

## Methods

For the present analysis we used data from a large prospective management study, the Christopher-study, including 3306 consecutive in-and outpatients, suspected of pulmonary embolism [8]. This study was performed in secondary care in the Netherlands between November 2002 and September 2004. It evaluated the safety of excluding PE by a sequential diagnostic work-up consisting of the dichotomous Wells CDR (cut-off ≤ 4), a quantitative D-dimer test and helical computer tomography (CT). Patients with a CDR indicating PE unlikely underwent D-dimer testing. Either the Vidas ELISA D-dimer test or the Tinaquant D-dimer test was used (cut-off ≤500 μg/l, combined sensitivity 97.8% and specificity 56.9%) and when normal, the diagnosis of PE was considered excluded. All other patients underwent helical CT. All patients were followed up for a period of 3 months to document the occurrence of subsequent symptomatic venous thrombo-embolism (VTE).

We used test characteristics of two qualitative POC D-dimer tests from a meta-analysis on the diagnostic accuracy of POC-D-dimer tests for excluding VTE [15].

1. SimpliRed D-dimer (sensitivity 85%, specificity 74%) is a semi qualitative test performed by mixing capillary or venous blood with a drop of test reagent in the test well. A positive result is defined as any visible agglutination within two minutes.

2. Simplify D-dimer (sensitivity 87%, specificity 62%) is a qualitative test and is performed by mixing 35 μl of capillary or venous blood with two drops of test reagent. A positive result is indicated by a visible pink-purple coloured line that forms at the test zone. The test can be read within 10 minutes.

To mimic a primary care setting we excluded all inpatients from the original cohort for the present analysis. As would be the case in primary care all patients with Wells CDR >4 needed imaging regardless of the D-dimer test result. Hence in these patients no additional D-dimer testing was performed.

Using the original Christopher-study data, we divided the remaining patients into groups according to their individual Wells-CDR scores with different cut-off values (Table 2). Within each group PE was excluded in patients with the combination of a Wells-CDR below the cut-off value and a negative D-dimer test result. Combining the prevalence of PE in each group with the sensitivity and specificity of the D-dimer test we calculated the theoretical failure-rate and the efficiency of the combined strategy in each clinical probability group.

**Table 2:** Results of failure-rate and efficiency in 2 scenarios at different cut-off values of the Wells-rule in comparison with results of the Christopher-study.

Wells	N=	Prevalence PE	SimpliRed: Sens 85% Spec 74%		Simplify: Sens 87% Spec 62%		Christopher 2006 Tinaquant/Vidas	
			
			Failure-rate (95% CI)	Efficiency	Failure-rate (95% CI)	Efficiency	Failure-rate (95% CI)	Efficiency
≤ 4	1876	12.0% (226/1876)	2.7% (1.9-3.8%)	46.5%	2.8% (1.9-3.9%)	38.9%	0.5% (0.2-1.2%)	35.0%

≤ 3	1772	11.3% (201/1772)	2.5% (1.7-3.6%)	44.2%	2.6% (1.7-3.8%)	37.0%	0.4% (0.1-1.1%)	34.1%

≤ 2	919	6.3% (58/919)	1.4% (0.6-2.6%)	23.9%	1.5% (0.6-2.9%)	20.1%	0.2% (0.0-1.0%)	19.8%

<2	915	6.3% (58/915)	1.4%(0.6-2.6%)	23.8%	1.5% (0.6-2.9%)	20.0%	0.2%(0.0-1.0%)	19.8%

≤ 1	611	4.6% (28/611)	0.9% (0.3-2.3%)	16.1%	1.1% (0.3-2.8%)	13.5%	0.0% (0.0-1.0%)	14.5%

0	559	4.3% (24/559)	1.0% (0.3-2.6%)	14.8%	0.9% (0.2-2.6%)	12.4%	0.0% (0.0-1.0%)	13.8%

N = Number of outpatients in different Wells clinical probability groups

CI = Confidence interval

Efficiency was defined as the proportion of all study patients, in whom PE was excluded (and thus would not need referral) based on a Wells-CDR below various cut-off values and a negative D-dimer test.

The failure rate was defined as the proportion of patients in whom PE was excluded based on a Wells-CDR below various cut-off values and a negative D-dimer test, with symptomatic and proven VTE during 3 months follow-up.

We considered ruling out PE safe if the failure rate was <2% with a maximum upper confidence limit of 2.7%, being the upper confidence limit of the three-month thrombo-embolic rate of patients suspected of PE but with a normal pulmonary angiography [16].

The 95% confidence intervals (CI) were calculated using Confidence Interval Analysis (CIA, version 1.0; Gardner MJ).

## Results

Of the total study population of 3306 in-and outpatients, 2701 were outpatients and included in this analysis. The prevalence of PE in the group of outpatients was 20.2%. (including the 3-months follow-up period). The prevalence of PE among patients with an unlikely clinical probability decreased with a decreasing CDR-cut-off value, ranging from 12.0% in patients with a Wells score ≤ 4 to 4.3% in patients with a Wells score of 0.

Table 2 shows the failure-rate and the efficiency at different cut-off values of the Wells-CDR in combination with the sensitivity and specificity of the D-dimer test. In the last column results of the outpatients obtained from the Christopher-study are depicted for comparison. In the Christopher-study PE could be excluded safely with a Wells-CDR cut-off value of ≤ 4 in 35.0% of the patients.

**Figure 1:**
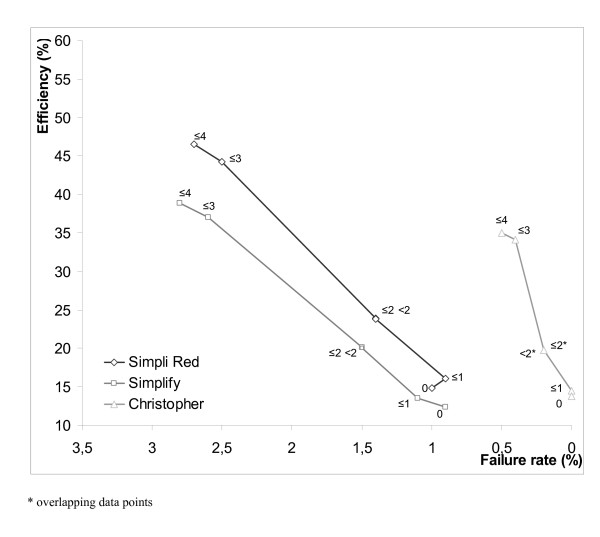
**Failure rate versus efficiency in 2 scenarios at different cut-off values of the Wells CDR in comparison with results from the Christopher-study**.

However the failure rate of 2.7% is exceeded in both qualitative POC D-dimer tests when combined with a Wells-CDR cut off value of ≤ 4. To meet the safety criteria (failure rate <2%, upper 95% CI <2.7%) the SimpliRed D-dimer test had to be combined with a Wells CDR-cut off value <2 and the Simplify D-dimer test with a Wells CDR-cut off value of 0. Using this strategy, the proportion of patients in whom PE might be excluded safely decreased to 23.8% with the SimpliRed D-dimer test and 12.4% with the Simplify D-dimer test. The dramatic loss in efficiency when using a lower Wells-CDR cut off is demonstrated in figure 1.

## Discussion

The current scenario-analysis determined the theoretical failure-rate and efficiency of a diagnostic strategy using the Wells CDR at different cut-off values combined with a qualitative POC D-dimer test for excluding PE in primary care. Excluding PE safely in primary care with a CDR and a point-of-care D-dimer test seems feasible. However, the strategy appeared to be safe only when the cut-off value of the Wells-CDR was lowered to <2 using the SimpliRed and 0 using the Simplify D-dimer test, respectively. Efficiency is considerably lower when using those cut-off values: the number of patients that need referral is 76.2% and 87.6% respectively, as compared to 65% with the Wells cut-off value of ≤ 4 in the Christopher-study.

Several aspects of this analysis require comment.

Firstly, we based the analysis on the test characteristics of two qualitative POC-D-dimer tests as reported in a diagnostic meta-analysis. In this meta-analysis most of the studies included patients suspected of DVT. Only six studies included patients with PE. However in a covariate analysis of studies with only DVT both the sensitivity and the specificity of the SimpliRed and the Simplify D-dimer test were essentially the same as in the overall analysis.

Secondly, several studies performed in secondary care (PE-prevalence ranging from 3.8-10%) show that a strategy using a CDR and a qualitative POC- D-dimer test can be used safely to exclude PE. Moreover these studies show a good efficiency ranging from 44-66% [7,10-13]. Wells et al were the first to show that the combination of Wells CDR <2 and a negative D-dimer test was safe to exclude PE. (prevalence 9.5%, failure rate 0.2%, efficiency 47%) [7]. According to Hogg and co-workers the Simplify D-dimer test alone was not sufficiently sensitive (sensitivity 81.8%, specificity 74.2%) to exclude PE in low-risk patients (prevalence PE 5.3%) presenting to the emergency department (ED) with pleuritic chest pain. However, when the Simplify D-dimer test was combined with a low-clinical probability Wells-rule the negative predictive value of the combined test was 99.3% (CI 97.4-99.9%): high enough to exclude PE safely [10]. Kline et al showed in low-risk ED-patients (prevalence PE 4.7%) that combination of a physician's unstructured estimate of pre-test probability of PE of <15% and a negative Simplify-D-dimer test excluded PE safely (sensitivity D-Dimer-test 80.6%, specificity 72.5%) [11]. In a primary care based management study sensitivity of the Simplify D-dimer test proved to be sufficient to exclude deep vein thrombosis (DVT) safely in patients with a low clinical probability. The relatively higher specificity, as compared to laboratory based quantitative D-dimer tests provided a good efficiency [14].

Although the sensitivity of the Simplify D-Dimer test in the studies of Hogg and Kline was only 81.8% and 80.6%, respectively, the negative predictive value of the combined strategy using a pre-test probability assessment and the Simplify D-Dimer test was high enough to exclude PE safely due to the low PE-prevalence in these studies.

Thirdly, a weak point of the analysis is that although we have excluded all in-patients the study-population is still not really a primary care population. The outpatients included in the Christopher-study are likely selectively biased as the primary care physician used his own judgement before referring the patient. In the Christopher-study the PE-prevalence was 20.2%. In daily practice when a primary care physician will use the Wells-CDR rule combined with a POC D-dimer test the prevalence of PE in suspected patients is expected to be lower which will improve the negative predictive value (and thereby safety and efficiency) of an exclusion strategy for PE in primary care.

Fourthly, we don't know how well the Wells CDR would perform in primary care. In secondary care the Wells rule is usually applied after routine blood tests, chest radiography and electrocardiography. The primary care physician is usually lacking this information and this will clearly influence the scoring of the subjective variable 'pulmonary embolism is as likely as or more likely than an alternative diagnosis'.

Fifthly, we know that the test characteristics of the POC-D-dimer test, unlike this scenario, are not fixed but are influenced by the prevalence of PE in the different Wells-groups. It is likely that the specificity of the D-dimer test will increase as the prevalence decreases. This might improve the negative predictive value of the strategy in primary care [17,18].

Sixthly, in this analysis the SimpliRED D-dimer assay was used which has certain limitations. It is known that this method may be associated with a risk for inadequate interpretation due to the fact that the results are based on a subjective interpretation of the presence or absence of agglutination [19]. This risk for inadequate interpretation will be enhanced by infrequent use of the assay. An average Dutch primary care physician will use a POC D-dimer assay for exclusion of PE only 3-5 times a year. However the physician will use the same assay also for exclusion of DVT [14]. We expect the Dutch primary care physician to apply the POC D-dimer test 12-15 times a year in both suspected PE-patients as DVT-patients. We think this will justify an adequate and reliable use of the assay.

Finally, although in scenario 2 (Simplify) the point estimate failure rate in Wells CDR < 2 is within the safety limits, the upper confidence limit exceeds 2.7%. Confidence intervals become larger with decreasing number of patients. It can be expected that with an increasing number of patients the proportion in the lower Wells-CDR score will be higher and the confidence interval will become narrower. Therefore scenario 2 might also be safe in Wells <2.

In secondary care, in a strategy using a more sensitive, quantitative D-dimer test, a cut-off value of Wells ≤ 4 is generally accepted as safe. Although the sensitivity of the POC qualitative D-dimer test is lower, the specificity of the test is higher and as a consequence efficiency is higher at the cost of safety. Recalibration of the Wells-rule for a primary care situation might overcome the safety problems.

## Conclusion

In this scenario-analysis we could exclude PE safely with a diagnostic strategy using the Wells CDR and a qualitative D-dimer test, albeit with only a moderate efficiency. A prospective study is needed to assess safety and efficiency of this strategy in a true primary care population. Recalibration of the Wells-rule or adaption of cut-off values might then be needed.

## Competing interests

The authors declare that they have no competing interests.

## Authors' contributions

RD, WL had full access to all of the data in the study and take responsibility for the integrity of the data and the accuracy of the data analysis. *Study concept and design*: HvW, WL. *Acquisition of data*: RD, WL. *Analysis and interpretation of data*: DT, HvW, WL. *Drafting of the manuscript*: HvW, WL. *Critical revision of the manuscript for important intellectual content*: DT, HB, HvW, RD, WL. *Statistical analysis*: WL. *Obtaining funding*: HvW. *Study supervision*: HvW, WL. All authors read and approved the final manuscript.

Data used in this document is openly available.

## Pre-publication history

The pre-publication history for this paper can be accessed here:

http://www.biomedcentral.com/1471-2296/11/64/prepub
